# Formation of planar hybrid lipid/polymer membranes anchored to an S-layer protein lattice by vesicle binding and rupture

**DOI:** 10.1080/1539445X.2019.1708753

**Published:** 2020-01-06

**Authors:** Christian Czernohlavek, Bernhard Schuster

**Affiliations:** Department of NanoBiotechnology, Institute for Synthetic Bioarchitectures, University of Natural Resources and Life Sciences, Vienna, Austria

**Keywords:** S-layer protein, lipid, polymer, membranes, quartz crystal microbalance with dissipation monitoring

## Abstract

Exploitation of biomolecular and biomimetic components on solid surfaces gain increasing importance for the design of stable functional platforms. The present study performed by quartz crystal microbalance with dissipation monitoring (QCM-D) reports on the formation of planar hybrid lipid/polymer membranes anchored to a crystalline surface (S-) layer protein lattice. In this approach, hybrid lipid/polymer vesicles were chemically bound to the S-layer protein lattice. Subsequently, to form a hybrid planar layer rupture and fusion was triggered either by (1) β– diketone – europium ion complex formation or (2) successive application of calcium ions, lowering the pH from 9 to 4, and the detergent CHAPS. As determined by QCM-D, method 1 resulted for a polymer content of 5% in a planar membrane with some imbedded intact vesicles, whereas method 2 succeeded in planar hybrid membranes with a polymer content of even up to 70%. These results provide evidence for the effective formation of planar lipid/polymer membranes varying in their composition on an S-layer protein lattice.

## Introduction

There are significant efforts to design synthetic membranes as mimics of biomembranes.^[1]^ The building blocks of bio-inspired systems constitute unique, predictable and tunable properties, diversity, and the possibility to fabricate ultra-thin two-dimensional structures in the square-micrometer range with an astonishing variety of functionalities at the nanoscale.^[2–7]^ Moreover, these natural building blocks like, e.g., lipids, proteins, and polymers self-assemble spontaneously into supramolecular structures.^[8–10]^ One of the most important template for bio-inspired architectures is the cell envelope structure of prokaryotes, which is a complex layered structure comprising of a cell membrane and in many cases of a monomolecular array of protein subunits forming the outermost surface layer (S-layer).^[4,9,11,12]^ The importance of cell membranes in biological systems as a barrier, preserver of the physical integrity of the cell and host for integral membrane proteins has prompted the development of membrane platforms that recapitulate fundamental aspects of membrane biology, especially the lipid bilayer environment.^[7,13,14]^ This environment is of utmost importance for integral membrane proteins like channels, proton pumps, and (G protein-coupled) receptors, which are responsible for carrying out important specific membrane functions.^[15–19]^

Immobilization of planar lipid mono- and bilayers on a solid support represent one of the most promising classes of model membranes, because these structures revealed a long shelf life and can be characterized by a wide range of surface-sensitive analytical techniques.^[20–23]^ Furthermore, these supported lipid membranes are increasingly able to mimic fundamental properties of natural cell membranes, including fluidity, electrical sealing, and hosting integral membrane proteins. The surface- (S-) layer protein lattice as tethering and anchoring structure in-between the solid support and the lipid membrane turned out to be a very straightforward approach as it significantly improves the integrity of the lipid membrane and it’s functioning as reconstitution matrix for integral membrane proteins.^[7,13,24–26]^

The challenge in biosensor development of integrating nonliving systems with biological ones led to problems associated with material interfacing and compatibility, as well as biological issues, such as viability and stability. Water is necessary to maintain the functionality of biological units, but has adverse effects on engineering components. Thus, a big challenge up to now is to extend significantly the longevity of membranes.^[7]^ An innovative approach is the generation of spherical mixed hybrid polymer/lipid membranes, which have so far carried out by the combined self-assembly of phospholipids and amphiphilic diblock copolymers.^[27–30]^ These hybrid vesicles revealed improved and modulated membrane bulk properties (e.g., permeability, toughness, elevated stability against mechanical stress, and air exposure).^[28]^ Based on these observations, the extension of the strategy to mix block copolymers with phospholipids for the creation of planar hybrid devices is anticipated to be a highly promising strategy to extend the longevity of planar bio-inspired membrane-mimicking architectures.^[30]^

**Figure 1:**
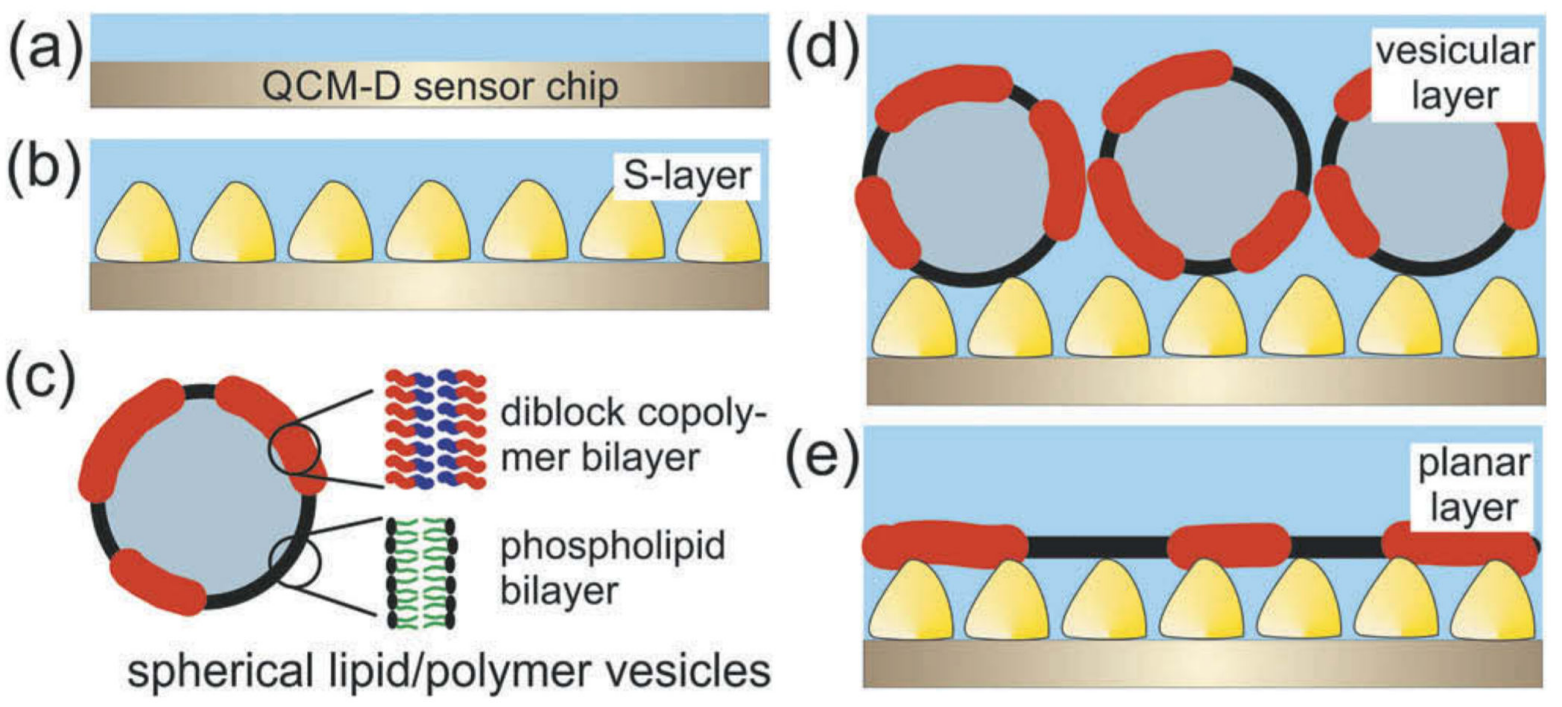
Schematic drawing of the formation of the mixed lipid/polymer layer on an S-layer lattice. Gold-coated QCM-D sensor crystal (a) onto which the S-layer protein SbpA from *Lysinibacillus sphaericus* CCM 2177 was recrystallized (b). The lipid/polymer vesicles (c) were added and chemically bound on the S-layer protein lattice to form a vesicular layer (d). Finally, two methods were applied in order to trigger the rupture and subsequent fusion of the hybrid vesicles to form a planar hybrid lipid/polymer layer on the S-layer lattice (e).

The present study is the first attempt to investigate the generation of planar hybrid lipid/polymer membranes with a certain polymer content by the vesicle fusion method on a crystalline S-layer lattice (Fig. 1). The polymer content varied from a low (5%–10%) to a high percentage (60%–70%) and the remaining part consisted of various phospholipids. The surface-sensitive, acoustical method quartz crystal microbalance with dissipation monitoring (QCM-D) measured (1) the S-layer protein recrystallization process (Fig. 1a,b)^[24,31]^, (2) the binding of the hybrid lipid/polymer vesicles (Fig. 1c,d), (3) the rupture and fusion process of the vesicles, and (4) eventually the formation of the anchored planar hybrid lipid/ polymer layer on the top of the S-layer lattice (Fig. 1e).

## Materials and Methods

### Materials and Procedures

Growth, cell wall preparation, and extraction of the SLP SbpA isolated from *Lysinibacillus sphaericus* CCM 2177 (Czech Collection of Microorganisms) were performed as previously described.^[32,33]^ SbpA stock solution was diluted with 0.5 mM Tris (pH = 9) containing 10 mM CaCl_2_ (buffer A) to a final concentration of 0.1 mg/mL.

400 mM 1-ethyl-3-(3-dimethylaminopropyl) carbodii-mide (EDC) and 200 mM sulfo-N-hydroxy-sulfo-succinimide sodium salt (S-NHS) (both Sigma-Aldrich) were stored as aliquots in 50 mM PBS buffer at pH 6.5 at −20°C. PBS buffer for the anchoring procedure was prepared by mixing monobasic and dibasic sodiumdihydrogenphosphate solutions in distinct ratios and brought to the desired pH by titration with HCl (Merck).

The diblock copolymer poly(butadiene-b-ethylene oxide) (poly butadiene block rich in 1,2 microstructure; BdEO) with a molecular weight of 1050 g/mol and a poly-dispersity index (PDI) of 1,09 was obtained from Polymer Source Inc., Dorval, Canada. The phospholipids 1,2-dimyristoyl-*sn*-glycerol-3-phosphoethanolamine (DMPE; M_w_ = 636 g/mol) and 1-palmitoyl-2-oleoyl-sn-glycerol-3-phosphocholine (POPC; M_w_ = 760 g/mol) were purchased from Avanti Polar Lipids. An amphiphilic β–diketone ligand (βDK) comprising of two C_16_ alkane chains as hydrophobic anchor and two short polyethylene glycol spacers onto which the βDK group is bound was used to facilitate the fusion of the vesicles.^[18]^ Two βDK groups preferentially incorporated in two adjacent vesicles form a complex with one Eu^3+^-ion, which triggers the fusion of the vesicles.^[34,35]^ The desired lipids were dissolved together with βDK and the polymer in chloroform. The latter was removed in a vacuum (50 mbar; 90 min) and by heating to 50°C. Subsequently, 200 mM sucrose in MilliQ water was added, and the hydrated lipid film was sonicated for 15 min to induce vesicle formation. Finally, the vesicles were extruded through a membrane filter with pores of 200 nm in diameter (Avestin, TX) to obtain unilamellar vesicles with a diameter of 160 nm (data not shown). The composition of the hybrid lipid/polymer vesicles is given in mole percent. However, the molar composition of lipid and polymer of the final hybrid vesicles might differ from the starting composition.^[28,36,37]^

QCM-D measurements were carried out with a Q-Sense E4 device (electronic unit) equipped with four standard flow modules (QSense, Biolin Scientific). The QSX 301 gold-covered crystal sensors have a fundamental frequency of 4.95 MHz (Q-Sense). After cleaning, the sensors were immediately mounted in the flow modules of E4. The flow rate was 0.1 mL/min. The shift in frequency (-Δ*f*) and dissipation (Δ*D*) were recorded using the Q-Soft 401 software. The presented results correspond to the fifth overtone. All experiments were performed at a temperature of 21°C ± 0.02°C. All given data are mean values of two independent measurements if not indicated other.

### Formation of the Layered Architectures

In a first step, the S-layer protein SbpA was recrystallized on the previously cleaned gold sensor surface by pumping the SbpA solution in the flow cell.^[38]^ Then, the sensor was rinsed with MilliQ water and the car-boxyl groups on the SbpA lattice were activated with the EDC/S-NHS solution (MilliQ water). After 10 min, a suspension containing the vesicles composed of DMPE (presenting terminal amino groups), βDK and BdEO and/or POPC was passed onto the S-layer lattice. In order to produce a gravimetric gradient, the cell was when rinsed with 200 mM glucose. For the fusion/ rupture of the vesicles, 1 mM EuCl_3_ (Sigma-Aldrich) in MilliQ water or in 200 mM glucose solution (MilliQ water) was passed over the vesicular layer. The complex formation of one Eu^3+^-ion with two βDK groups located at adjacent vesicles comprising low polymer content caused in many cases the rupture and fusion of the bound vesicles.^[38]^ Vesicles comprising a high polymer content did nor open and fuse with each other and hence, the vesicular layer was incubated with 200 mM CaCl_2_ solution (MilliQ water), citrate buffer (pH 4.0), and/or with a 0.2% solution of the detergent 3-[(3-cholamidopropyl) dimethyl-ammonio] -1-propane-sulfonate (CHAPS). If not other indicated, the presented data are the mean values of two independent measurements.

## Results and Discussion

### Binding of Hybrid Lipid/polymer Vesicles on the S-layer Lattice

**Figure 2:**
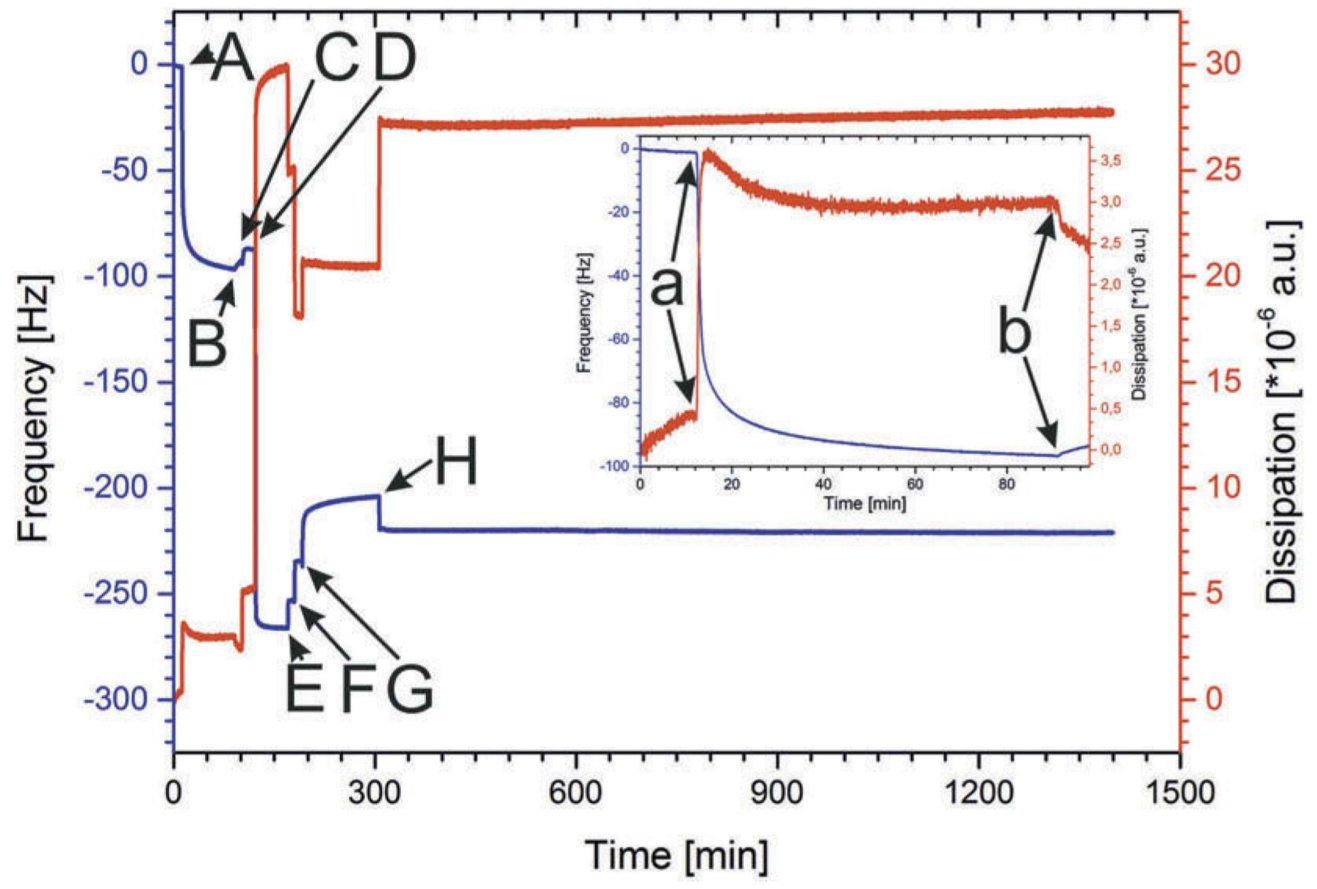
Shift in frequency (blue) and dissipation (red) during the formation process of the S-layer lattice (SbpA, from Lysinibacillus sphaericus) and the subsequent binding of hybrid lipid/polymer vesicles. (a): injection of the SbpA solution, (b): rinsing with MilliQ water, (c): injection of EDC/S-NHS, (d): injection of the lipid/polymer vesicles (POPC:DMPE: BdEO = 50:25:25), (e): rinsing with 200 mM glucose, (f): rinsing with milliQ water, (g): rinsing with buffer A, (h): stop rinsing. Insert: Magnification of the S-layer protein recrystallization. The SbpA was passed over the sensor surface (a) and subsequently the S-layer was rinsed with MilliQ water (b).

The S-layer protein SbpA was passed after 12 min over the gold surface of the QCM-D sensor (Fig. 1a; Fig. 2a, Insert a). The frequency immediately dropped down and after approximately 50 min almost the maximum in -Δ*f* was reached (Fig. 2). After approximately 70–90 min, the recrystallization of SbpA to form a coherent monolayer on the gold surface was finished (Fig. 1b, Fig. 2b, Insert). The average for the final shift in frequency was calculated to −90.8 ± 4.6 Hz (n = 9). The Δ*D* signal showed immediately after injection of SbpA a strong increase to a maximum of 3.6*10^−6^ a.u. and finally the Δ*D* value leveled off to an average final shift value of 2.58 ± 0.38*10^−6^ a.u. (n = 9). The shape of the curves and final shift of both frequency and dissipation are well known to correspond to the formation of a crystalline S-layer lattice on the gold-coated sensor surface (Fig. 2, Insert).^[7,24,26,31]^ The S-layer lattice may act not only as an anchoring scaffold for the membrane, but also as spacer structure between the lipid/ polymer membrane and the solid support, and functions as ion reservoir.^[7]^

The aim of this experiment was to determine if hybrid lipid/polymer vesicles bind via the terminal amino groups of the DMPE lipids on the underlying S-layer lattice (Fig. 1c). Hence, the incubation with EDC/S-NHS activated the carboxyl groups of the SbpA by forming reactive ester groups (Fig. 2c). Subsequently, without any intermediate rinsing step, the lipid/polymer vesicles comprising of 50% POPC, 25% DMPE, and 25% BdEO, were injected and incubated with the activated S-layer lattice (Fig. 2d). As the vesicles contained 200 mM sucrose, they sedimented by gravity force down to the SbpA-covered surface. In order to get a balanced ion strength, the flow cell was rinsed with 200 mM glucose (Fig. 2e). Finally, the vesicular layer on SbpA was rinsed with MilliQ water (Fig. 2f) and buffer A (Fig. 2g) to obtain the same comparable environmental conditions as for the recrystallized S-layer lattice. After stopping the rinsing process (Fig. 2h), -Δ*f* decreased and Δ*D* increased and remained stable over a period of 1100 min (Fig. 2). Thus, this experiment clearly showed that it was possible to bind the hybrid lipid/polymer vesicles in a highly stable manner onto the S-layer lattice (Fig. 1d). The shift in frequency is with approximately −230 Hz very pronounced and indicated the formation of a vesicular layer on the S-layer lattice, where S-layer protein, lipid polymer, but also the entrapped and coupled water/ sucrose contributed to the mass on the sensor surface. The high dissipation of approximately 28*10^−6^ a.u., however, indicated the vesicular layer to be very soft and viscoelastic. As these experiments were successful, the challenge to form planar hybrid membranes by the opening of S-layer protein-bound vesicles was accepted.

### Formation of Hybrid Lipid/polymer Layer with Low Polymer Content on the S-layer Lattice

**Figure 3:**
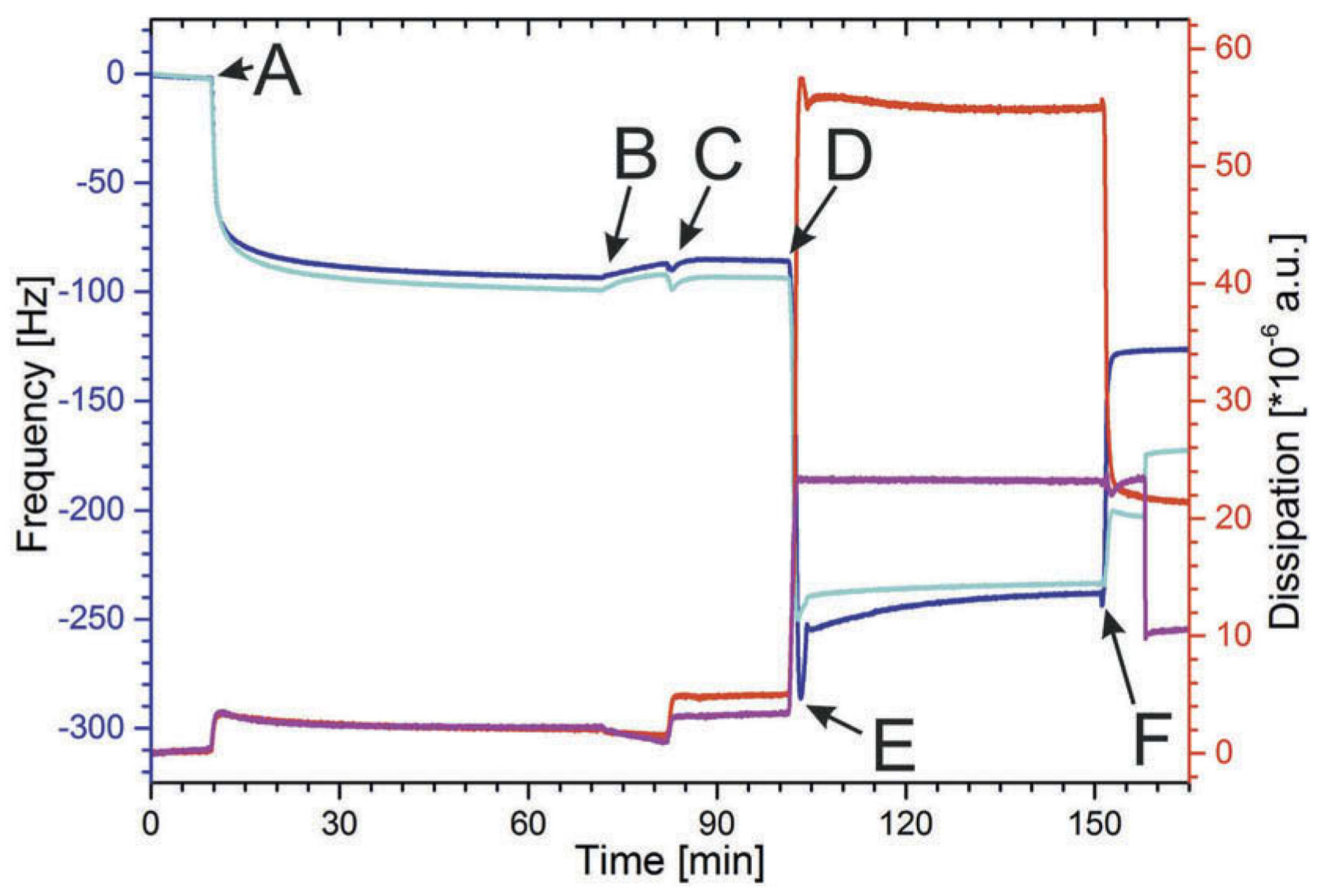
Shift in frequency (blue, cyan) and dissipation (red, magenta) during the formation of the S-layer supported lipid membrane and the subsequent binding of hybrid lipid/polymer vesicles comprising of vesicles POPC:DMPE:BdEO:βDK = 74: 20:5:1 (blue, red) and POPC:DMPE:BdEO: βDK = 69:20:10:1 (cyan, magenta). (a) injection of SbpA protein solution; (b) rinsing with MilliQ water; (c) activation of S-layer with EDC/ S-NHS; (d) addition of hybrid lipid/polymer vesicles; (e) Stopping the pump and incubation of SbpA layer with vesicles; F) addition 1 mM EuCl_3_ to trigger fusion/rupture of the vesicles.

As shown in Figure 3, SbpA was injected at point A and the recrystallization was finished after approximately 70 min. At point B, Figure 3, the SbpA lattice was rinsed with MilliQ water and subsequently activated by adding EDC/S-NHS (Fig. 3, C) as described in section 3.1. After activation, the lipid/polymer vesicles (POPC:DMPE:BdEO:βDK = 74:20:5:1 and 69:20:10:1) were passed on the proteinaceous surface (Fig. 3, point D), which caused a dramatic change in both -Δ*f* and Δ*D*. After rinsing (Fig. 3, E), the frequency decreased to a value of approximately −250 Hz, which corresponds to the binding of a huge mass comprising of lipids, polymer and vesicle-entrapped and -coupled water. The -Δ*f* value is for both hybrid vesicles comprising of either 5% or 10% BdEO similar. The Δ*D* value. however, is for the hybrid vesicles with the lower BdEO content (5%) significantly higher (55*10^−6^ a.u.) compared the hybrid vesicles with the higher BdEO content (10%; 23*10^−6^ a.u.). This result provides evidence that the vesicles containing a higher polymer content formed a stiffer vesicular layer on the S-layer lattice.

After the binding of the hybrid polymer/lipid vesicles onto the S-layer lattice (Fig. 1d), the EuCl_3_ solution was passed over the vesicular layer to induce fusion and rupture of the bound vesicles (Fig. 3, F). As previously mentioned,^[38]^ the complex formation of one Eu^3+^-ion with two βDK groups located at adjacent vesicles should trigger rupture the vesicles and induce the formation of a planar layer on the S-layer lattice (Fig. 1e). The vesicles with the higher BdEO content of 10% showed an increase of -Δ*f* to finally ~175 Hz and a decrease in Δ*D* to ~10*10^−6^ a.u. This result indicates the formation of a rigid (Δ*D*) but still vesicular layer, because the difference of -Δ*f* to the plain S-layer lattice (−100 Hz) is −75 Hz. This value is much too high to indicate the formation of a planar layer. According to the literature, a planar lipid bilayer on an S-layer lattice gives rise to a shift in frequency of −23 ± 4 Hz.^[38]^ Interestingly, BdEO has almost the same area per molecule than POPC (data not shown) but the molar weight is for BdEO by 38% higher. Hence, it is conceivable that the shift in frequency is per se larger for a hybrid lipid/polymer membrane compared to a planar membrane comprising only of POPC.^[29,38]^ However, the polymer content of the here formed membrane is with 10% BdEO low and hence, one can not conclude that a layer with Δ*f* of −75 Hz corresponds to a planar layer, as the theoretical additional mass contributed from BdEO can be calculated to less than 1 Hz. Thus, one has to conclude that the vesicles comprising 10% BdEO could only be partly triggered to rupture and fuse and a significant proportion of intact vesicles are bound to the S-layer lattice.

The vesicles with the lower BdEO content of 5% showed again an immediate increase of Δ*f* to a final value of ~-128 Hz and a decrease in Δ*D* to finally ~21*10^−6^ a.u. (Table 1). This result of Δ*D* indicates the formation of a very soft hybrid lipid/polymer layer. However, because the difference to the plain S-layer lattice (−94 Hz) is −34 Hz (Table 1) few intact vesicles might be embedded in the planar layer. The measured Δ*D* value of ~21*10^−6^ a.u. supported also the presence of vesicles embedded in a hybrid planar layer, making the overall layer more viscoelastic.

One may speculate that the higher polymer content of 10% instead of 5% might result in more stable lipid/ polymer vesicles and/or may shield the βDK ligands from the Eu^3+^-ions. Thus, these vesicles with 10% BdEO can not be ruptured by the βDK – Eu^3+^-ion complex formation. Consequential vesicles comprising a higher BdEO content have not been studied by method 1.

The schematic drawing in Figure 1 indicated a membrane with separated domains of lipid and polymer, which is only one possible structure among many. Nevertheless, vesicles composed of lipid/polymer mixtures showed this structural feature.^[28]^ However, structural features like domain formation of the presently formed planar hybrid lipid/polymer layer has not yet been determined as QCM-D is not the appropriate technique. Hence, further investigation, e.g., by microscopical and/or spectroscopical techniques have to be performed to elucidate this issue in more detail.

### Formation of Hybrid Lipid/polymer Layer with High Polymer Content on the S-layer Lattice

**Figure 4:**
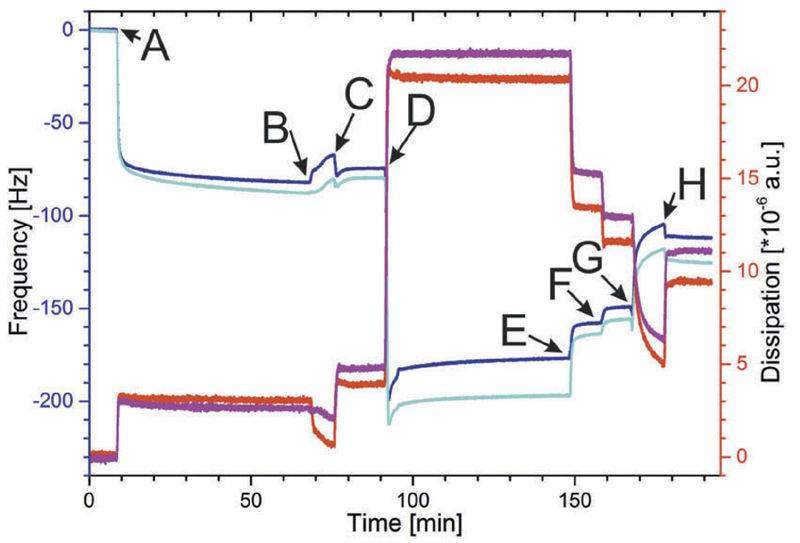
Shift in frequency (blue, cyan) and dissipation (red, magenta) during the formation of the S-layer supported lipid membrane and the subsequent binding of hybrid lipid/polymer vesicles comprising of vesicles DMPE:BdEO = 30:70 (blue, red) and DMPE: BdEO = 40:60 (cyan, magenta). (a) injection of SbpA protein solution; (b) rinsing with MilliQ water; (c) activation of S-layer with EDC/ S-NHS; (d) addition of hybrid lipid/polymer vesicles; (e) rinsing with a CaCl_2_ solution; (f) rinsing with citrate buffer, pH 4.0; (g) rinsing with 0.2% CHAPS; (h) rinsing with 200 mM glucose.

In this set of experiments, hybrid lipid/polymer vesicles comprising of only DMPE and BdEO at a ratio of 40:60 and 30:70 were generated. No βDK was added to the hybrid lipid/polymer vesicles because previous experiments (see section 3.2) showed that the rupture/fusion process triggered by βDK was limited to vesicles comprising of only 5% BdEO as vesicles with 10% BdEO remained intact. The challenge was to elucidate whether it will be possible to push the BdEO content to a high percentage or not. As a certain proportion of DMPE was necessary for the binding of the hybrid vesicles onto the S-layer lattice, no POPC was present in these hybrid vesicles. The initial procedure (recrystallization and activation of SbpA, binding of the hybrid vesicles; Fig. 4a–d) was the same as described in sections 3.1 and 3.2.

As in section 3.2, after the activation process, the polymer/lipid vesicles were passed on the proteinaceous surface (Fig. 4, point D), which caused a dramatic shift in both -Δ*f* and Δ*D*. The frequency decreased to a value of approximately −200 Hz and 180 Hz for the DMPE:BdEO = 40:60 and 30:70, respectively. Both data correspond to the binding of a huge mass (i.e., DMPE, BdEO, and vesicle-entrapped and -coupled water), on the SbpA-covered sensor surface (Fig. 1d). The Δ*D* value is for both hybrid vesicular layers with 21.7*10^−6^ a.u. and 20.3*10^−6^ a.u. for the DMPE:BdEO = 40:60 and 30:70, respectively similar. Moreover, these Δ*D* values are close to the value of 23*10^−6^ a.u., which was found for the layer comprising of POPC: DMPE:BdEO:βDK = 69:20:10:1. This result provides evidence that vesicular DMPE/BdEO layers with similar physical properties like, e.g., mass, thickness (indicated by -Δ*f*), and stiffness (indicated by Δ*D*) have been formed on the S-layer protein-covered sensors.

In Figure 4, point E, the layer comprising of hybrid lipid/polymer vesicles was rinsed with a 200 mM CaCl_2_ solution. Calcium ions stabilize the permeability of cell membranes.^[39]^ Hence, a first attempt was to weaken the membrane of the hybrid lipid/polymer vesicles by calcium ions. The rinsing with CaCl_2_ solution caused for both vesicular DMPE/BdEO layers an increase in -Δ*f* and decrease in Δ*D*. This result indicated that some of the vesicles might have ruptured. However, the lipid/polymer layer on the S-layer lattice was comprised most probably of regions with a planar membrane and many bound intact vesicles. In a next attempt to form a planar hybrid lipid/polymer layer, the system was rinsed with a citrated buffer, pH = 4.0 (Fig. 4, point F). Lowering of the pH value might induce a pH-driven morphological transition of the BdEO structure.^[40]^ Shifting the pH value from 9.0 to 4.0 caused similar to the calcium treatment for both vesicular DMPE/BdEO layers an increase in -Δ*f* and decrease in Δ*D*. This effect is not so pronounced than the effect caused by the calcium ions. However, the result indicated that at least few vesicles might have ruptured by lowering the pH value. In a further attempt to form a planar hybrid lipid/polymer layer, the system was rinsed with 0.2% CHAPS (Fig. 4, point G). CHAPS, a zwitterionic detergent and derivative of the bile salts is widely used in membrane studies.^[41]^ It is a “facial” detergent, having a hydrophilic side and a hydrophobic back.^[42]^ Rinsing with CHAPS caused, similar to the calcium treatment and lowering the pH value for both vesicular DMPE/BdEO layers an increase in the -Δ*f* value and decrease in Δ*D*. This effect was the most pronounced one and caused a shift in -Δ*f* after rinsing with glucose to a value of −120 Hz and −110 Hz for the DMPE:BdEO = 40:60 and 30:70, respectively (Fig. 4, point H). The initial recrystallization of SbpA revealed a shift in frequency of −90 Hz and −84 Hz for the DMPE:BdEO = 40:60 and 30:70, respectively. Thus, the difference in frequency between the S-layer lattice and the formed hybrid layer was determined to be −30 Hz and −26 Hz for DMPE:BdEO = 40:60 and 30:70, respectively (Table 1). The formed lipid/polymer membrane showed compared to the pure lipid membrane resting an S-layer lattice, slightly higher shifts in frequency.^[16]^

Rinsing with CHAPS caused the Δ*D* values significantly dropping down to a higher extent than observed for the calcium treatment and lowering the pH and were finally determined to 11.1*10^−6^ a.u. and 9.5*10^−6^ a.u. for the DMPE:BdEO = 40:60 and 30:70, respectively (Table 1). The shift in dissipation for the DMPE/BdEO planar membranes are in very good accordance with previously published data for pure lipid membranes bound to an S-layer lattice.^[16]^ If one cancels out the polymer content of 5% where intact vesicles are embedded in the planar hybrid layer, the Δ*D* values decrease with increasing polymer content (Table 1). Hence, this result provides evidence that an increasing amount of BdEO makes the membrane stiffer and less viscoelastic. However, this ‘stiffening effect’ might occur only on a high polymer percentage.

As QDM-D measures the bound mass averaged over the whole sensor surface, further investigation by, e.g., spectro-scopical and/or electrochemical techniques have to be performed. These techniques might elucidate in detail whether a closed planar hybrid lipid/polymer layer has been formed or the layer consisted of patches of bound vesicles, ruptured and fused planar membranes and areas where no polymer/ lipid material has bound to the S-layer lattice.

## Conclusions

The aim of the present study was to pioneer the reproducible formation of a planar hybrid lipid/polymer membrane resting on the crystalline layer comprising of SbpA. For this purpose, a closed S-layer lattice was recrystallized on the surface of gold-coated sensor crystals. In this new approach, hybrid lipid/polymer vesicles were bound on the SbpA lattice and two different methods were applied to rupture the vesicles, which should subsequently fuse to form a planar hybrid layer on the S-layer lattice.

First, hybrid vesicles containing βDK ligands were generated and bound to the SbpA lattice. Rinsing with a Eu^3+^-solution triggered the formation of a soft but planar layer on the S-layer lattice. However, this technique resulted for a polymer content of 5% in a planar hybrid membrane with few imbedded intact vesicles. Second, hybrid planar lipid/polymer membranes with a high polymer content of up to 70% were formed on an S-layer lattice by incubation of the bound hybrid vesicles with a Ca^2+^-solution, lowering the pH, and finally by rinsing with CHAPS. Indeed, the values derived from QCM-D measurements for both hybrid DMPE/BdEO layer bound to the S-layer lattice are in very good agreement with previously published data for planar lipid membranes anchored to an underlying S-layer lattice.

It became evident by the present study that planar hybrid lipid/polymer membranes can be formed in a wide range of polymer percentage. Hence, further studies on biophysical properties, in particular stability parameters like, e.g., longevity, mechanical stress, osmotic pressure, resistance to chemicals and enzymes, etc. are feasible in the future.

The capabilities of supported lipid membranes have already opened the door to biotechnology applications in medicine, diagnostics, sensor systems, environmental monitoring and energy storage. As hybrid lipid/polymer vesicles showed an enhanced mechanical stability and permeability barrier, planar S-layer supported hybrid membrane hold great promise to design and generate robust and versatile bioinspired architectures. Integration of these planar S-layer supported hybrid lipid/polymer membrane with nonliving systems constitute promising bioinspired systems to bridge the worlds of conventional engineering and biology and could strongly contribute to the development of both. On the one hand, more powerful tools to study, handle, and engineer these natural units could be obtained while, on the other hand, it may be possible to fabricate novel sequencing, high-throughput screening, and sensory system devices.

## Figures and Tables

**Table 1:** Summary of the QCM-D data for planar hybrid lipid/ polymer membranes.

% of BdEO	-Δ*f* [Hz]	Δ*D* [*10^6^ a.u.]	Rupture/fusion triggered by
0	23	11.5	βDK
5	34	21.0	βDK
60	30	11.1	Ca^2+^, pH shift, CHAPS
70	26	9.5	Ca^2+^, pH shift, CHAPS
